# Time-series and risk-adjusted control charts

**DOI:** 10.1186/1748-5908-8-S1-S5

**Published:** 2013-04-19

**Authors:** Patricia S Groves, Caitlin W Brennan, Michael E Matheny

**Affiliations:** 1 Veterans Affairs National Quality Scholars Program, Iowa City VA Medical Center, Iowa City, Iowa, 52246, USA; 2 University of Iowa College of Nursing, Iowa City, Iowa, 52242, USA; 3 Veterans Affairs National Quality Scholars Program, Louis Stokes Cleveland VA Medical Center, Cleveland, Ohio, 44106, USA; 4 Frances Payne Bolton School of Nursing, Case Western Reserve University, Cleveland, Ohio, 44106, USA; 5 Center for Organization, Leadership, and Management Research, VA Boston Healthcare System, Boston, Massachusetts, 02130, USA; 6 Geriatrics Research Education and Clinical Care Center, VA Tennessee Valley Healthcare System, Nashville, Tennessee, 37212, USA; 7 Departments of Medicine, Bioinformatics, and Biostatistics, Vanderbilt University Medical Center, Nashville, Tennessee, 37232, USA

## Presentation

There are several statistical process control (SPC) methods used in industry that can be applied in healthcare. However, as noted in the earlier discussion by Toulany et al. regarding quasi-experimental designs for quality improvement research, several considerations must be taken into account when adapting these methods for the complex, high-risk healthcare arena. Industrial methods should be adjusted for (a) heterogeneity at the patient level, including illness type, individualized care, and demographics, (b) heterogeneity at the process level, including geographical and longitudinal clinical care variation, (c) lack of pre-existing standards of comparison for new products or processes, and (d) the critical difference between statistical variation and acceptable clinical risk. Potential methods for successful adaptation of industrial SPC methods for healthcare monitoring and improvement include (a) converting periodic data into cumulative charts to increase detection of trends and (b) addressing heterogeneity through risk adjustment, using a prediction model or propensity score matching. These adjustments tend to inflate Type I errors, however, due to repeated measurements. Thus, the Sequential Probability Ratio Testing (SPRT) method may be of particular use [1]. SPRT uses the more commonly available retrospective control data, accounts for repeated measurements, utilizes risk adjustment, and incorporates both alpha and beta error into the formal framework [2,3]. The upper control limit is the desired odds ratio, as determined by the hypothesis.

Industrial SPC methods assume process homogeneity and that the outcome rate from the population establishes the threshold for detecting changes in the process. Using these techniques to analyze processes in healthcare often requires addressing the risk and complexities inherent in healthcare in order to obtain meaningful results. As with any data-driven project, the clinical question and limitations of the available data drive the selection of the patient cohort, SPC method, risk adjustment framework, alerting thresholds, and the interpretation of clinical significance. However, regardless of the SPC method used and the risk-adjustment framework, it is important to realize that performance of the risk adjustment model drives the overall result; thus understanding the strengths and weaknesses of each particular model is critical to clinical interpretations. In addition, detecting adverse outcomes over a long period requires recalibrating the model over time to adjust for systematic changes in clinical care. Finally, all signals detected using these methods require root cause analyses (RCA) and sensitivity analyses as they are hypothesis-generating, not confirming.

## Commentary

The use of time series and risk-adjusted control charts are increasingly common methods of presenting and analyzing data related to quality improvement initiatives. Once the domain of industries that were concerned with consistent performance and production of mechanical outputs, SPC techniques are now being used to monitor clinical outcomes, benchmark data in quality registries, and provide surveillance of new medical products and adverse events. Despite the large amounts of data that are often analyzed, the near-real time surveillance of outcomes using these techniques allows quick detection of special cause variation. In turn, interpretation of special causes may result in quick identification of clinically meaningful efficacy or conversely, concerning signals that safety is at risk. After a negative special cause is detected, an RCA can be used to identify likely risk factors and determine the real signal of interest requiring amelioration amongst the clinical and statistical noise. For example, SPC techniques in combination with an RCA were used to identify a link between retroperitoneal hemorrhage and using vascular closure devices with high femoral access in percutaneous coronary interventions. This finding was used to prompt educational interventions and modify clinical practice [4].

Multiple sources of data are available for conducting quality improvement research or initiatives utilizing SPC techniques, both locally and on a large scale. These sources include electronic medical records (EMRs), regional and national data registries, and clinical trial registries. Additionally, the appropriate SPC method can be used both for relatively rare events and for more frequently occurring events, increasing their utility for a range of settings and outcomes of interest.

Thus increasing sophistication and adaptation of proven industrial SPC methods, particularly in terms of risk-adjustment and measurement of rare outcomes, have provided additional tools to both those conducting local quality improvement initiatives and investigators who rely on the validity of quality registries to conduct research. This has the potential to increase the immediate clinical impact of quality improvement work as well as the dissemination of interpretable data and research findings. These methods are increasing in use within the healthcare domain, but remain an open area of methodology research as some complexities of healthcare data, such as repeated measurements of the same patient or institution, require further adaptation of these methods.

## Recommendations

There are four key requirements and recommendations for making greater use of these SPC methods. First, additional infrastructure for collecting valid and reliable data is needed. The data required to drive these methods is facilitated by the structured data entry and collection from the EHR. This may include building basic EHR infrastructure; greater interoperability between EHRs; restructuring of EHRs, including templates for capturing specific variants; and greater use of natural language processing to capture potentially relevant details from free text and dictated notes. Second, collaboration between statisticians, registry experts, healthcare informaticists, and clinicians is required to address the complexity and heterogeneity inherent in data registries. The expertise of each is needed to extract, risk-adjust, and interpret the data required for answering the right question in an understandable way. Third, the limited amount of funds available to improve data infrastructure, support expertise, and collect and maintain the necessary data may necessitate restructuring of the payment system, potentially at both the state and national level. Because all payers, as well as patients and healthcare facilities, benefit from improved care, new methods of sharing savings and reforming payments could support advancement of these methods. Finally, as SPC methods continue to evolve and the infrastructure needed to support them grows, use of key techniques such as time-series and risk-adjusted control charts should be integrated into the clinical and graduate education of healthcare professionals, assuring their availability and accessibility for healthcare improvement.
